# Caring for patients with multimorbidity: moral distress and life satisfaction among doctors and nurses in Portugal

**DOI:** 10.7717/peerj.21230

**Published:** 2026-04-29

**Authors:** Filipe Prazeres, Rui Fernandes

**Affiliations:** 1 Faculty of Health Sciences, University of Beira Interior, Covilhã, Portugal; 2 Family Health Unit Beira Ria, Gafanha da Nazaré, Portugal; 3 RISE-Health, Universidade da Beira Interior, Covilhã, Portugal; 4 Palliative Care Unit, Portuguese Institute of Oncology, Porto, Portugal

**Keywords:** Multimorbidity, Moral distress, Life satisfaction, Doctor, Nurse, Healthcare providers

## Abstract

**Background:**

The prevalence of multimorbidity (MM), defined as the co-occurrence of two or more chronic conditions in an individual, presents complex challenges for healthcare providers (HCPs). In Portugal, nearly half of the general population experiences MM, intensifying demands on HCPs. Doctors and nurses often encounter fragmented care pathways, inadequate guidelines, and frequent ethical dilemmas, which can lead to moral distress (MD). MD arises when HCPs are unable to act in accordance with their ethical beliefs due to institutional barriers, and it has consequences for well-being and employee tenure. This study aims to examine whether MM-related clinical work and life satisfaction are associated with MD among HCPs who regularly care for patients with MM.

**Methods:**

Cross-sectional study surveyed doctors and nurses employed in hospital and non-hospital healthcare facilities in Portugal between August and October 2024. Data were collected using an anonymous electronic questionnaire including validated instruments: the Portuguese version of Measure of Moral Distress for Healthcare Professionals (MMD-HP) and the Satisfaction With Life Scale (SWLS). Additional sociodemographic and work-related information was gathered, including sex, age, marital status, professional role, workplace setting, number of years working with patients with MM (professional experience), and the number of patients with MM seen per week (clinical workload). Associations were examined using linear multiple regression, with the significance level set at 0.05.

**Results:**

A total of 340 HCPs participated, mostly women (83.2%), nurses (66.8%), and professionals with more than 10 years of experience caring for patients with MM (75.6%). The median MD (MMD-HP) score was 128 (Q1, Q3: 73, 182); median life satisfaction (SWLS) score was 24 (Q1, Q3: 18, 28). MD was negatively correlated with life satisfaction, indicating that lower life satisfaction was associated with higher MD. Higher MD levels were observed in HCPs under 35 years compared with those over 50 (*p* = 0.010). HCPs with more than 10 years of experience caring for patients with MM reported significantly lower MD (*p* = 0.022). A higher MM-related clinical workload was also associated with greater MD (*p* = 0.003). HCPs currently considering leaving their position due to MD reported significantly higher MD and lower life satisfaction (both *p* < 0.0001). In multivariable analysis, MM-related clinical workload and life satisfaction remained significant predictors of MD.

**Discussion:**

MD was associated with higher MM-related clinical workload and lower life satisfaction. Younger and less experienced HPCs appeared particularly vulnerable. The reliability of these findings should be interpreted with caution due to the use of composite scores and the observational study design . The results reinforce concerns about the impact of MD on intentions to leave healthcare positions and emphasize the need for institutional support/interventions that address workload and promote well-being.

## Introduction

The growing prevalence of multimorbidity, frequently defined as the co-occurrence of two or more chronic conditions in an individual (Ho et al., 2021), presents a significant challenge to healthcare systems worldwide (Pearson-Stuttard, Ezzati & Gregg, 2019). Portugal has a mostly tax-funded, universal healthcare system, where general practitioners act as gatekeepers and care is delivered through both public and contracted private providers (Fronteira, Augusto & Maresso, 2025). In Portugal, multimorbidity has been reported in up to 48.9% of the general population (Pascoa et al., 2022), with estimates indicating a 13.1% increase in prevalence by 2050 (Laires & Perelman, 2019). Population ageing and rising chronic disease burden increase the clinical and organizational complexity of care delivery. Patients with multimorbidity often require individualized, long-term, and interdisciplinary care that extends beyond the scope of single-disease models (Spiers et al., 2023; Tinetti, Fried & Boyd, 2012). Healthcare providers (HCPs), including doctors and nurses, are tasked with navigating inadequate clinical guidelines (Cohen-Stavi et al., 2020; Sinnott et al., 2013), fragmented care pathways (Prior et al., 2023; Sinnott et al., 2013), and competing treatment priorities within healthcare systems that are not structured to deliver integrated chronic care, such as the Portuguese system. These structural and clinical complexities may give rise to ethically challenging situations in routine practice.

In such contexts, HCPs often face situations in which they know what would be right for the patient but are constrained by institutional policies, consultation time pressures, resource limitations, and inconsistent treatment recommendations (Prazeres & Santiago, 2016; Prazeres, Simões & Lomas, 2021). These experiences can lead to moral distress (MD), a psychological phenomenon that occurs when individuals are unable to act according to their ethical beliefs due to institutional barriers, as originally described by Jameton (1984). More recent work, however, has expanded the concept to include a broader range of morally challenging situations and recognizes MD as a response to various types of moral conflict and constraints (Fourie, 2015). The conceptual boundaries of MD, particularly regarding the respective roles of moral uncertainty and perceived constraint, remain the subject of scholarly debate. While acknowledging these ongoing discussions, the present study does not aim to resolve competing conceptual positions. Rather, we adopt an operational definition suitable for empirical investigation in health services research.

Although the present study focuses on MD, it is important to acknowledge that caring for patients with multimorbidity often involves situations of moral uncertainty, where the ethically optimal course of action is not immediately clear due to competing health priorities or ambiguous guidelines. Such uncertainty may co-occur with MD, as HCPs who identify what they believe to be the right action may still face constraints in implementing it. However, in the present framework, epistemic uncertainty alone is not considered sufficient to constitute MD; rather, MD is understood as arising when a healthcare professional forms a moral judgment yet perceives barriers to acting upon it. This interplay situates MD within the broader ethical complexity of multimorbidity care while preserving its operational focus as a measurable psychological construct.

First conceptualized in the nursing profession, MD is currently recognized as a significant problem affecting healthcare providers across various disciplines (Ulrich, Hamric & Grady, 2010; Pina, 2019), and reports of it have increased in the context of the COVID-19 pandemic restrictions (Quek et al., 2022). MD is associated with feelings of guilt, anger, frustration, and helplessness (Wiegand & Funk, 2012; Wilkinson, 1987), and can result in psychological and physical impact, compassion fatigue, professional dissatisfaction, burnout, reduced employee tenure, and compromised patient care (Aljabery et al., 2024; Austin, Saylor & Finley, 2017; Morley et al., 2017; Pina, 2019; Quek et al., 2022).

MD is prevalent in contexts involving high-stakes clinical decision-making under conditions of systemic constraint, particularly when actions exceed deeply held moral beliefs, as may occur in end-of-life care, intensive care units, and oncology (Mehlis et al., 2018; Mills & Cortezzo, 2020). Despite MD being well-studied in acute and critical care settings, no quantitative studies have examined its predictors and impact among HCPs providing clinical care for patients with multimorbidity. This study therefore represents the first empirical investigation of these associations in this context. Unlike acute care scenarios, the ethical challenges associated with multimorbidity are often prolonged and tied to clinical complexity and systemic limitations in delivering a person-oriented approach (Molinaro et al., 2024; Upshur, 2016). HCPs may be compelled to prioritize certain conditions over others, make trade-offs that compromise patients’ quality of life, or carry out treatment plans that they believe are futile or misaligned with patients’ interests (Currie & Laing, 2023), all while lacking the time, resources, or institutional authority to change existing care structures (Weiner, 2020). These situations are marked not only by clinical complexity but also by competing normative commitments, potentially contributing to sustained moral strain rather than isolated episodes of moral conflict.

At the same time, there is increasing recognition of the importance of healthcare professionals’ overall well-being. Life satisfaction is defined as a person’s subjective evaluation of the quality of their life as a whole (Sousa & Lyubomirsky, 2001), encompassing factors such as happiness, health, meaning, character, social relationships, and financial security (Padgett et al., 2025). In healthcare settings, physician dissatisfaction and poor well-being can compromise care quality, leading to medical errors and reduced patient outcomes (Casalino & Crosson, 2015). Conversely, high life satisfaction may serve as a protective factor, buffering against the emotional and psychological consequences of prolonged occupational stress and moral strain (Duarte et al., 2022; Haber et al., 2013; Martins et al., 2022; Padmanabhanunni, Pretorius & Isaacs, 2023). Life satisfaction may be associated with the intensity of MD experienced in ethically challenging contexts. Individuals with higher life satisfaction may draw on broader psychosocial resources, demonstrate more adaptive appraisal processes, or exhibit greater emotional regulation (Kaynak, Denizci Nazligul & Cengil, 2024; Kim et al., 2016; Martin, Collie & Holliman, 2024; Mittal, 2020; Potoczny, Herzog-Krzywoszańska & Krzywoszański, 2025) when navigating moral tensions.

Multimorbidity-related clinical work represents a structural exposure variable that may increase the frequency and intensity of ethically challenging situations. Greater involvement in multimorbidity-related complex care may therefore be associated with higher levels of MD. Examining both structural (work-related) and individual (life satisfaction) factors allows for a more comprehensive understanding of predictors of MD in this context.

Moreover, differences in professional roles may shape how MD is experienced. Nurses, who often provide prolonged bedside care and serve as advocates for patients, may face distinct ethical pressures compared to doctors, who are more likely to be involved in decision-making and resource allocation (Choi et al., 2023). Exploring these differences may help clarify how MD manifests across professional groups in multimorbidity care.

Given these considerations, the present study investigates whether multimorbidity-related clinical work and life satisfaction are associated with MD among HCPs who regularly care for patients with multimorbidity. By examining these associations, the study aims to contribute to a more nuanced understanding of MD in chronic care settings and to inform institutional strategies that support healthcare professionals’ well-being in the face of increasing clinical complexity.

## Materials & Methods

This study employed a cross-sectional design. The sample consisted of doctors and nurses employed in both hospital and non-hospital health facilities across Portugal. A minimum sample size between 196 and 384 participants was estimated based on a total population of 142,195 doctors and nurses in Portugal (National Institute of Statistics, 2024), using a 95% confidence level, a 5–7% margin of error, and assuming the most conservative scenario (population proportion = 50%).

Data were collected between August and October 2024 *via* an anonymous electronic survey. Recruitment was conducted through relevant medical groups on social media and the professional networks of the research team, and participants were able to share the survey with their own contacts, resulting in a non-random, convenience sample. This approach was intended to reach a wider and more diverse range of HCPs than would have been feasible with random or institution-based recruitment. Potential participants received a link to the survey, which was hosted on Google Forms. To enhance participation, two reminder messages were distributed. No incentives were provided.

Prior to data collection, ethical approval was obtained from the University of Beira Interior Ethics Board (CE-UBI-Pj-2024-033). The study adhered to the ethical principles outlined in the Declaration of Helsinki. Informed electronic consent was obtained from all participants before they began the questionnaire. All responses were submitted anonymously.

The questionnaire was structured to be completed in under 15 min and was pretested with 20 doctors and nurses to ensure clarity and ease of understanding. It included multiple-choice, numeric and scaled-response items, and several components. All questions were mandatory, and participants were required to respond to every item before submitting the questionnaire.

First, participants provided sociodemographic and work-related information, including their sex, age, marital status, professional role, and workplace setting, number of years working with patients with multimorbidity, and number of patients with multimorbidity seen per week.

The number of years working with patients with multimorbidity was used as an indicator of professional experience. Because meaningful exposure to multimorbidity care requires sustained clinical experience, and participants with very limited experience were underrepresented in our sample, the variable was dichotomized into “10 years or less” and “more than 10 years”. This categorization ensured adequate group sizes for statistical analysis while preserving a clear distinction between less- and more-experienced HCPs. In medical and clinical research, a 10-year threshold is commonly used to differentiate early-career physicians from senior or more experienced clinicians (Dugani et al., 2020; Karabiçak & Arslan, 2025; Li et al., 2016). The number of patients with multimorbidity seen per week by HCPs was used as a proxy indicator of multimorbidity-related clinical workload and was collapsed into a dichotomous variable by splitting the data at the median value to form “high” and “low” groups, as no clinical cut-offs were available.

Next, the Portuguese version of the Measure of Moral Distress for Healthcare Professionals (MMD-HP) scale (Dias, Teixeira & Antunes, 2022) was used to assess the frequency and intensity of morally distressing situations in healthcare settings (Epstein et al., 2019). This instrument includes 27 items, each rated on a 0–4 scale for both frequency (0 = never, 4 = very frequently) and distress level (0 = none, 4 = very distressing). A composite score is calculated by multiplying frequency and distress ratings for each item and summing the resulting products to measure current levels of MD. The resulting score will have a range of 0–432 (Epstein et al., 2019). There are also two final questions: “Have you ever left or considered leaving a clinical position due to moral distress?” (No, I have never considered leaving or left a position; Yes, I considered leaving but did not leave; Yes, I left a position) and “Are you considering leaving your position now due to moral distress?” (Yes; No). The MMD-HP scale used is freely available online at: https://www.actamedicaportuguesa.com/revista/index.php/amp/article/view/16531/6528 and the authors have permission to use this instrument from the copyright holders. It showed a good internal consistency with a Cronbach’s α = 0.943, 95% CI [0.934–0.951], in the present sample, confirming the good psychometric properties of the scale.

Finally, the Satisfaction With Life Scale (SWLS), a psychometrically robust and widely used 5-item scale that assesses global life satisfaction (Diener et al., 1985), was administered in its Portuguese version (Laranjeira, 2009; Neto, 1993). Participants rated their agreement with each item on a 7-point Likert scale, ranging from 1 (strongly disagree) to 7 (strongly agree), with higher scores indicating greater life satisfaction. A total score is obtained by summing the five items, ranging from 5 to 35 points. It showed a good internal consistency with a Cronbach’s α = 0.908, 95% CI [0.892–0.923] in the present sample, confirming the good psychometric properties of the scale.

Prior to analysis, all data were checked for entry errors and missing values. Outliers and influential observations were examined, and all assumptions of the regression models were verified. Data were analyzed using IBM SPSS v. 25 (IBM Corp., Armonk, NY, USA), with the significance level set at 0.05. Categorical variables are presented as absolute and relative frequencies (n, %), and continuous variables as medians with interquartile ranges (Med; Q1, Q3). The univariate analysis for quantitative variables used a Mann–Whitney test for variables with two response options and Kruskal–Wallis test for three or more. Association between two categorical variables was tested by Chi Square test and Fisher test. Associations between predictors and MD were examined using linear regression. As this is an empirical study without *a priori* theoretical assumptions, variables with *p* < 0.20 in the univariate analysis were entered into the multivariable model. This approach allows inclusion of potentially relevant predictors while maintaining model parsimony.

## Results

Table 1 shows the overall responses from the 340 participants. The majority are women (83.2%), aged between 35 and 50 years (45%), married or in a stable union (65.9%), nurses (66.8%), working in non-hospital healthcare facilities (50.3%), have been working for more than 10 years with patients with multimorbidity (75.6%), and reported a high clinical workload related to patients with multimorbidity (52.4%).

**Table 1:** Main characteristics of the study participants (*n* = 340).

	n	%
**Sex**		
Male	57	16.8
Female	283	83.2
**Age (years)**		
Under 35	56	16.5
35 to 50	153	45.0
Over 50	131	38.5
**Marital status**		
Married/or in a stable relationship	224	65.9
Single/Separated/Divorced/Widowed	116	34.1
**Professional role**		
Doctor	113	33.2
Nurse	227	66.8
**Workplace setting**		
Hospital	169	49.7
Non-hospital healthcare facility	171	50.3
**Number of years working with patients with multimorbidity**		
10 or less	83	24.4
More than 10	257	75.6
**Multimorbidity clinical workload**		
High	178	52.4
Low	162	47.6

The median number of patients with multimorbidity seen per week (Q1 = first quartile, Q3 = third quartile) was 30 (15, 50) patients.

For MD (MMD-HP), scores ranged from 0 to 432 points. The median score was 128 (Q1, Q3: 73, 182) points. For life satisfaction (SWLS), scores ranged from 5 to 35 points. The median score was 24 (Q1, Q3: 18, 28) points (Table 2).

**Table 2:** Medians (Q1–Q3) for HCPs’ MD (MMD-HP) and life satisfaction (SWLS) (*n* = 340).

	Minimum–Maximum	Median (Q1–Q3)
MD (MMD-HP)	0–432	128 (73–182)
Life satisfaction (SWLS)	5–35	24 (18–28)

A Spearman’s correlation was conducted to evaluate the relationship between life satisfaction and MD. A significant negative correlation was found (r_s_ = −0.250, *p* < 0.0001).

Table 3 shows that no significant associations were found between MD and sex (*p* = 0.060), marital status (*p* = 0.621), professional role (*p* = 0.779), or workplace setting (*p* = 0.663). However, HCPs under the age of 35 reported significantly higher levels of MD compared to those over 50 years old (*p* = 0.010). The 35–50 age group exhibited intermediate levels, with scores similar to both younger and older participants. HCPs with more than 10 years of experience working with patients with multimorbidity reported significantly lower MD levels (*p* = 0.022). In contrast, those managing a high multimorbidity-related clinical workload reported significantly higher MD compared to those with a lower workload (*p* = 0.003). Furthermore, HCPs who had never considered leaving a clinical position due to MD reported significantly lower MD scores than those who had either considered leaving or had actually left a position (*p* < 0.0001). Those currently considering leaving their positions also reported significantly higher MD levels than those who are not (*p* < 0.0001).

**Table 3:** Comparison of MD levels with the other variables.

	Median	Q1–Q3	*p*-value^*^
**Sex**			
Male	100	54–165	0.060
Female	132	77–185	
**Age (years)**			
Under 35 (a)	150	102–209	
35 to 50 (a, b)	128	83–191	0.010
Over 50 (b)	114	60–171	
**Marital status**			
Married/or in a stable relationship	132	75–184	0.621
Single/Separated/Divorced/Widowed	121	70–178	
**Professional role**			
Doctor	133	65–175	0.779
Nurse	127	82–184	
**Workplace setting**			
Hospital	129	66–190	0.663
Non-hospital healthcare facility	125	81–172	
**Number of years working with patients with multimorbidity**			
10 or less	145	98–210	0.022
More than 10	116	69–180	
**Multimorbidity clinical workload**			
High	138	91–205	0.003
Low	117	61–167	
**Have you ever left or considered leaving a clinical position due to moral distress?**			
No, I have never considered leaving or left a position (a)	90	47–130	<0.0001
Yes, I considered leaving but did not leave (b)	151	106–208	
Yes, I left a position (b)	169	113–265	
**Are you considering leaving your position now due to moral distress?**			
No	116	65–168	<0.0001
Yes	169	115–267	

**Notes.**

* Mann–Whitney test for variables with two response options and Kruskal–Wallis test for three response options. In the latter case, the difference is found using the Bonferroni test, represented by letters. When the letters are the same, the groups are equal.

**Figure 1:**
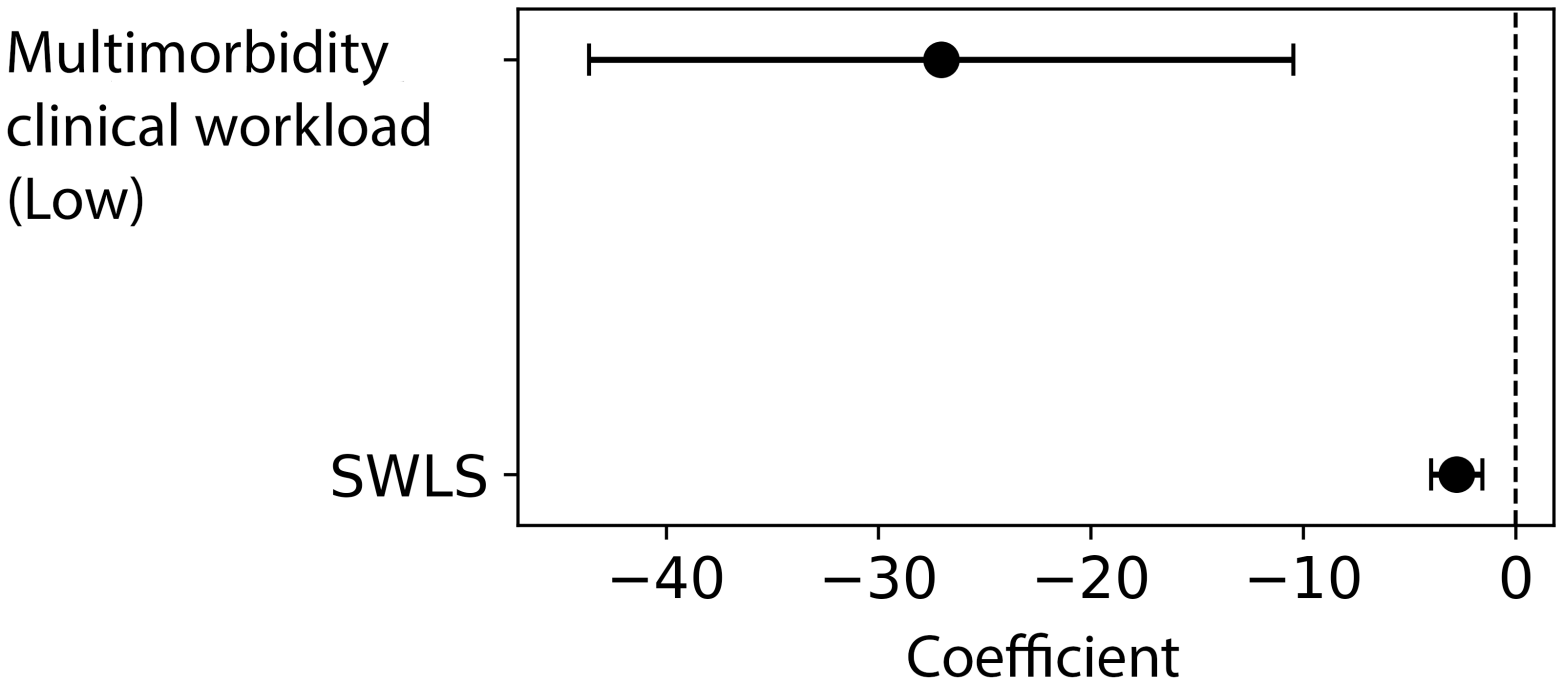
Forest plot of multivariable linear regression results examining associations between multimorbidity-related clinical workload, life satisfaction (SWLS), and moral distress.

In the multivariable analysis, two variables remained significant and were retained in the final model: multimorbidity-related clinical workload and life satisfaction. A lower multimorbidity-related clinical workload was associated with reduced MD scores. Additionally, higher life satisfaction was associated with lower MD scores (Table 4). These associations are visually summarized in Fig. 1, which displays the regression coefficients and 95% confidence intervals.

**Table 4:** Predictors identified on multivariable analysis according to the outcome variable (MD).

**Outcome – MD levels**				
**Predictor variables in the model**	**Coef.**	**Std. error**	**95% CI**	***p*-value**
Multimorbidity clinical workload (Low)	−27.041	8.457	−43.616; −10.465	0.001
SWLS	−2.780	0.616	−3.988; −1.572	<0.001
(Constant)	212.156	15.091	182.577; 241.735	<0.001

Table 5 shows that there is no significant difference between the characteristics of the group not considering leaving their position and the group considering this possibility, except in the evaluation of MD and life satisfaction: MD was significantly lower in the group not intending to leave the position (*p* < 0.0001), whereas life satisfaction was significantly higher in this group (*p* < 0.0001).

**Table 5:** Association of variables with the intention to leave the position.

	**Are you considering leaving your position now due to moral distress?**		
	No (*n* = 276)	Yes (*n* = 64)	*p*-value^*^
**Sex**			
Male	44 (15.9%)	13 (20.3%)	0.399
Female	232 (84.1%)	51 (79.7%)	
**Age (years)**			
Under 35	44 (15.9%)	12 (18.8%)	0.822
35 to 50	126 (45.7%)	27 (42.2%)	
Over 50	106 (38.4%)	25 (39.1%)	
**Marital status**			
Married/or in a stable relationship	179 (64.9%)	45 (70.3%)	0.407
Single/Separated/Divorced/Widowed	97 (35.1%)	19 (29.7%)	
**Professional role**			
Doctor	89 (32.2%)	24 (37.5%)	0.421
Nurse	187 (67.8%)	40 (62.5%)	
**Workplace setting**			
Hospital	137 (49.6%)	32 (50%)	0.958
Non-hospital healthcare facility	139 (50.4%)	32 (50%)	
**Number of years working with patients with multimorbidity**			
10 or less	66 (23.9%)	17 (26.6%)	0.657
More than 10	210 (76.1%)	47 (73.4%)	
**Multimorbidity clinical workload**			
High	138 (50%)	40 (62.5%)	0.071
Low	138 (50%)	24 (37.5%)	
	Median (Q1–Q3)	Median (Q1–Q3)	
MD (MMD-HP)	116 (65–168)	169 (115–267)	<0.0001
Life satisfaction (SWLS)	25 (20–28)	20 (13–25)	<0.0001

**Notes.**

* Mann Whitney test for MD and SWLS. For the other variables, Chi-Square test.

## Discussion

The present study provides the first insights into the perceived MD and life satisfaction among HCPs caring for patients with multimorbidity. Overall, HCPs reported moderate levels of MD. However, since the MMD-HP scale is relatively recent and lacks established cut-off points for classifying MD as high or low (Epstein et al., 2019), these results should be interpreted with caution. In contrast, life satisfaction levels were relatively high, with scores exceeding the neutral point of the SWLS scale (20 points) and falling within the upper range of “slightly satisfied” (21–25 points) (Pavot & Diener, 2008).

Internationally, MD is a significant and recurring concern across multiple clinical disciplines. Intensive care unit (ICU) nurses report moderate levels of MD in their practice (Qu et al., 2026), and pediatric trainees caring for patients with neurologic conditions appear particularly vulnerable (Di Domenico et al., 2025). Genetic counselors who provide inpatient care also experience MD in the course of their work (Vermeire et al., 2025). Additionally, evidence suggests that MD is pervasive in radiology (Dave et al., 2025), underscoring that this phenomenon spans diverse healthcare roles and settings rather than being confined to a single specialty.

A key finding was the significant negative correlation between life satisfaction and MD, suggesting that lower life satisfaction may be associated with heightened experiences of MD. This finding is broadly consistent with previous work that characterizes life satisfaction as a protective factor against burnout, such as studies on Portuguese nurses during the COVID-19 pandemic (Martins et al., 2022), medical students in northern Portugal during the same pandemic period (Duarte et al., 2022), hospital physicians in Israel (Haber et al., 2013), and South African higher education students facing stressful life events (Padmanabhanunni, Pretorius & Isaacs, 2023). Although the cross-sectional design of the present study limits causal interpretations, the observed findings are consistent with the hypothesis that higher life satisfaction may serve as a protective factor, potentially buffering HCPs against experiences of MD.

Notably, this study revealed that younger HCPs (under 35) reported significantly higher levels of MD compared to their older counterparts. This may suggest that limited experience in navigating complex clinical and ethical scenarios contributes to greater MD. This interpretation aligns with previous research showing that a lack of experience, low self-confidence, and insufficient ethical knowledge increased MD among newly graduated nurses working in public and private hospitals in Turkey (Kovanci & Atli Ozbas, 2025). Similarly, age has been found to be inversely associated with MD among non-physician health professionals in 13 intensive care units in British Columbia, Canada (Dodek et al., 2016). In the present study, HCPs with less than 10 years of experience managing patients with multimorbidity also reported higher MD scores, highlighting the potential buffering effect of professional experience in mitigating MD.

Workload also emerged as a critical factor, as participants who reported a high clinical workload related to multimorbidity experienced significantly greater levels of MD compared to those with lower demands. This finding supports previous research linking resource strain and workload intensity to MD in clinicians (Bradshaw, 2019) and social workers (Mänttäri-van der Kuip, 2015). These results underscore how persistent pressures, such as the complexity of managing patients with multimorbidity, can exacerbate ethical tensions, leaving HCPs feeling unable to provide care that aligns with their values.

In the multivariate analysis, multimorbidity-related clinical workload and life satisfaction remained significantly associated with MD, highlighting their potential as priority areas for intervention. Efforts to enhance life satisfaction, along with improvements in staffing, resource allocation, and team-based care models, may help reduce MD and support HCPs well-being, particularly in high-demand multimorbidity settings.

Surprisingly, no significant associations were found between MD and sex, marital status, professional role, or healthcare setting, suggesting that MD in multimorbidity care may be a widespread experience, irrespective of demographic and professional boundaries. However, this finding should be interpreted with caution, as the sample was predominantly composed of women and nurses, which may limit the generalizability of the results. Moreover, other studies have reported different patterns, nurses generally experience higher levels of MD compared to physicians, and professionals working in hospital settings report greater MD than those in community care (Giannetta et al., 2021). Additionally, those working in ICUs have been found to experience higher MD than professionals in pediatric or non-ICU settings (Whitehead et al., 2015).

HCPs who were considering leaving their positions due to MD unsurprisingly reported significantly higher MD scores and lower life satisfaction. These findings reinforce concerns that unresolved MD may contribute to the intention to leave one’s position, particularly in multimorbidity-related care contexts.

Despite these findings, some limitations warrant mention. The cross-sectional design limits causal interpretation, and the reliance on self-reported measures introduces the possibility of response bias. Some variables, such as multimorbidity-related clinical workload and professional experience, were dichotomized, which may reduce variability and statistical power. The study did not measure certain potential confounders, such as overall workload, institutional factors, or individual coping strategies, which could influence MD. Additionally, the sample was skewed toward female and nursing professionals, and the non-random, convenience recruitment method may limit the representativeness of the findings and, therefore, their generalizability. However, this approach also allowed us to reach a wider and more diverse sample of HCPs than would have been feasible with random or institution-based recruitment. Measurement instruments, while validated, include items that may be subjective or double-barrelled (Oelhafen, Monteverde & Trachsel, 2025). The use of a composite score represents an additional limitation. From a psychometric perspective, aggregating items assumes that the construct is sufficiently unidimensional, which was not formally tested in the present study. Although internal consistency, as assessed by Cronbach’s alpha, was high, this does not provide evidence of unidimensionality or full construct validity (Sijtsma, 2009). Consequently, the reliability of the composite measure should be interpreted with caution. Future research should explore item-level or subscale analyses to examine more specific dimensions of MD. Finally, the recruitment method may have introduced self-selection bias, as individuals already familiar with the concepts of multimorbidity and/or MD may have been more inclined to participate; while difficult to avoid, this should be considered when interpreting the findings. Future research could benefit from longitudinal designs, larger and more diverse samples, and qualitative approaches to better understand the nuanced drivers of MD in multimorbidity care.

## Conclusions

The present study highlights the associations between multimorbidity-related clinical workload, life satisfaction, and MD among HCPs. Findings suggest that MD is related to both life satisfaction and workload in the context of multimorbidity care. However, the reliability of these findings should be interpreted with caution due to the use of composite scores and the observational study design. The results are correlational, and we cannot infer causality or claim that MD is unique to multimorbidity-related care.

These findings suggest that interventions aimed at supporting HCPs well-being and managing workload demands may play a role in reducing MD and promoting workforce retention in multimorbidity-related care contexts. Future research should examine MD across different workload contexts and care domains to better understand its broader determinants.

## Supplemental Information

10.7717/peerj.21230/supp-1 Supplemental Information 1 Raw Data

10.7717/peerj.21230/supp-2 Supplemental Information 2 STROBE checklist

10.7717/peerj.21230/supp-3 Supplemental Information 3 Questionnaire (Portuguese)

10.7717/peerj.21230/supp-4 Supplemental Information 4 Questionnaire (English)

10.7717/peerj.21230/supp-5 Supplemental Information 5 Codebook

## Additional Information and Declarations

### Data Availability

The following information was supplied regarding data availability: SPSS data file.
