# Supplementary material for: Caring for patients with multimorbidity: moral distress and life satisfaction among doctors and nurses in Portugal
Source: PeerJ. 2026 Apr 29;14:e21230. doi: 10.7717/peerj.21230 (PMC13135333; doi:10.7717/peerj.21230)
Supplement: Supplemental Information 3 [file peerj-14-21230-s003.docx]

**Sexo:**

- Masculino
- Feminino
- Prefere não responder

**Idade** (anos):

**Estado Civil:**

- Solteiro
- Casado/União de facto
- Separado/Divorciado
- Viúvo

**Profissão:**

- médico(a)
- enfermeiro(a)

**Local de trabalho:**

- Hospital
- Estabelecimento de saúde não hospitalar

**Tempo de trabalho com doentes com multimorbilidade (2 ou mais doenças crónicas):**

- menor ou igual a 1 ano
- maior que 1 ano e menor ou igual a 5 anos
- maior que 5 anos e menor ou igual a 10 anos
- maior que 10 anos

**Qual julga ser o número de doentes com multimorbilidade que observa numa semana típica de trabalho?**

**Portuguese version of MMD-HP**

**Medida do Sofrimento Moral – Profissionais de Saúde**

O sofrimento moral acontece quando os profissionais não podem realizar o que acreditam ser ações eticamente apropriadas devido a constrangimentos ou barreiras. Este inquérito apresenta situações que acontecem na prática clínica. Se alguma vez experienciou estas situações, estas podem ou não ter sido moralmente difíceis. Por favor, indique com que frequência vivenciou a situação descrita em cada item e quantifique o quanto lhe provocou sofrimento moral. Se nunca vivenciou determinada situação, selecione “0” (nunca) para a frequência. Mesmo que não tenha vivenciado a situação, por favor, indique quanto **sofrimento moral** lhe poderia ter causado se ela tivesse ocorrido na sua prática.

**Note que responderá a cada item, assinalando a sua resposta nas duas dimensões: Frequência e Nível de Sofrimento.**

|  | **Frequência** | | | | | **Nível de Sofrimento** | | | | |
| --- | --- | --- | --- | --- | --- | --- | --- | --- | --- | --- |
|  | Nunca Muito  frequentemente | | | | | Nenhum Muito  intenso | | | | |
|  | 0 | 1 | 2 | 3 | 4 | 0 | 1 | 2 | 3 | 4 |
| 1. Testemunhar profissionais de saúde a dar “falsas esperanças” a um doente ou família. |  |  |  |  |  |  |  |  |  |  |
| 2. Dar seguimento a um tratamento agressivo por insistência familiar, mesmo acreditando não ser no melhor interesse do doente. |  |  |  |  |  |  |  |  |  |  |
| 3. Sentir-se pressionado a pedir ou cumprir um pedido de exames e tratamentos que considera serem desnecessários ou inadequados. |  |  |  |  |  |  |  |  |  |  |
| 4. Estar impossibilitado de prestar os melhores cuidados possíveis  devido a pressões da administração ou seguradoras para reduzir custos. |  |  |  |  |  |  |  |  |  |  |
| 5. Continuar a prestar um tratamento agressivo a uma pessoa que muito provavelmente morrerá, independentemente deste tratamento, quando ninguém toma a decisão de o suspender. |  |  |  |  |  |  |  |  |  |  |
| 6. Ser pressionado para não atuar quando verifico que um médico, enfermeiro ou outro membro da equipa cometeu um erro médico e  não o reporta. |  |  |  |  |  |  |  |  |  |  |
| 7. Ser solicitado para cuidar de doentes não me sentindo qualificado para o fazer. |  |  |  |  |  |  |  |  |  |  |
| 8. Participar em cuidados que causam sofrimento desnecessário ou não aliviam adequadamente a dor ou outros sintomas. |  |  |  |  |  |  |  |  |  |  |
| 9. Observar os cuidados ao doente serem afetados devido à falta da sua continuidade. |  |  |  |  |  |  |  |  |  |  |

| 10. Cumprir com o pedido de um médico ou familiar para não discutir o prognóstico com o doente/família. |  |  |  |  |  |  |  |  |  |  |
| --- | --- | --- | --- | --- | --- | --- | --- | --- | --- | --- |
| 11. Testemunhar a violação de um padrão de prática profissional ou de princípios éticos e não se sentir suficientemente apoiado para a denunciar. |  |  |  |  |  |  |  |  |  |  |
| 12. Participar em cuidados com os quais não concordo, mas ter de fazê- lo por medo de litígio. |  |  |  |  |  |  |  |  |  |  |
| 13. Ser-me exigido trabalhar com outros membros da equipa de saúde que não são tão competentes quanto os cuidados ao doente o exigem. |  |  |  |  |  |  |  |  |  |  |
| 14. Testemunhar prestação de cuidados ao doente com baixa qualidade devido a má comunicação na equipa. |  |  |  |  |  |  |  |  |  |  |
| 15. Sentir-se pressionado para ignorar situações nas quais não foi dada informação suficiente aos doentes de modo a garantir o consentimento informado. |  |  |  |  |  |  |  |  |  |  |
| 16. Ser solicitado a cuidar de mais doentes do que aqueles que me é possível cuidar em segurança. |  |  |  |  |  |  |  |  |  |  |
| 17. Vivenciar comprometimento dos cuidados ao doente devido a falta de recursos/equipamento/capacidade de camas. |  |  |  |  |  |  |  |  |  |  |
| 18. Vivenciar falta de ação ou apoio administrativo para um problema que compromete os cuidados ao doente. |  |  |  |  |  |  |  |  |  |  |
| 19. Sentir os cuidados ao doente comprometidos por excesso de burocracia. |  |  |  |  |  |  |  |  |  |  |
| 20. Temer represálias se falar com franqueza. |  |  |  |  |  |  |  |  |  |  |
| 21. Sentir insegurança/intimidação/bullying por parte dos meus próprios colegas. |  |  |  |  |  |  |  |  |  |  |
| 22. Ter de trabalhar com doentes/familiares abusivos que comprometem a qualidade dos cuidados. |  |  |  |  |  |  |  |  |  |  |
| 23. Sentir a obrigação de sobrevalorizar tarefas e medidas de produtividade ou qualidade em detrimento dos cuidados ao doente. |  |  |  |  |  |  |  |  |  |  |
| 24. Ter de cuidar de doentes que têm planos de tratamento ambíguos ou inconsistentes ou que não têm objetivos de cuidados definidos. |  |  |  |  |  |  |  |  |  |  |
| 25. Trabalhar em hierarquias de poder na minha equipa, unidade ou instituição que comprometem os cuidados ao doente. |  |  |  |  |  |  |  |  |  |  |
| 26. Integrar uma equipa que transmite mensagens inconsistentes ao doente/família. |  |  |  |  |  |  |  |  |  |  |
| 27. Trabalhar com membros da equipa que não tratam doentes vulneráveis ou estigmatizados com dignidade e respeito. |  |  |  |  |  |  |  |  |  |  |
| Se houver outras situações nas quais tenha sentido sofrimento moral, por favor, escreva-as e avalie-as aqui: |  |  |  |  |  |  |  |  |  |  |
|  |  |  |  |  |  |  |  |  |  |  |
|  |  |  |  |  |  |  |  |  |  |  |

**Já alguma vez deixou ou considerou deixar o seu cargo devido ao sofrimento moral?**

- Não, nunca o fiz nem considerei fazê-lo.
- Sim, considerei fazê-lo, mas não o fiz.
- Sim, já o fiz.

**Está a considerar deixar o seu cargo devido ao sofrimento moral?**

- Sim
- Não

**Encontra a seguir cinco afirmações com as quais pode concordar ou discordar. Utilizando a escala de 1 a 7 abaixo indicada, refira o seu grau de acordo com cada item colocando o número apropriado na linha que precede cada um deles. Procure ser sincero nas respostas que vai dar. Eis a escala de 7 pontos:**

| 1 – totalmente em desacordo |  | | | | | | |
| --- | --- | --- | --- | --- | --- | --- | --- |
| 2 – em desacordo |  |  |  |  |  |  |  |
| 3 – mais ou menos em desacordo |  |  |  |  |  |  |  |
| 4 – nem de acordo nem em desacordo |  |  |  |  |  |  |  |
| 5 – mais ou menos de acordo |  |  |  |  |  |  |  |
| 6 – de acordo |  |  |  |  |  |  |  |
| 7 – totalmente de acordo |  |  |  |  |  |  |  |
| 1. Em muitos aspetos, a minha vida aproxima-se dos meus ideais. | 1 | 2 | 3 | 4 | 5 | 6 | 7 |
| 2. As minhas condições de vida são excelentes. | 1 | 2 | 3 | 4 | 5 | 6 | 7 |
| 3. Estou satisfeito com a minha vida. | 1 | 2 | 3 | 4 | 5 | 6 | 7 |
| 4. Até agora, consegui obter aquilo que era importante na vida. | 1 | 2 | 3 | 4 | 5 | 6 | 7 |
| 5. Se pudesse viver a minha vida de novo, não alteraria pratica- | 1 | 2 | 3 | 4 | 5 | 6 | 7 |

menta nada.

**Obrigado pela participação.**
