# Supplementary material for: Caring for patients with multimorbidity: moral distress and life satisfaction among doctors and nurses in Portugal
Source: PeerJ. 2026 Apr 29;14:e21230. doi: 10.7717/peerj.21230 (PMC13135333; doi:10.7717/peerj.21230)
Supplement: Supplemental Information 4 [file peerj-14-21230-s004.docx]

**Sex:**

- Masculine
- Feminine
- Prefer not to answer

**Age**(years):

**Marital status:**

- Single
- Married/De facto union
- Separated/Divorced
- Widower

**Profession:**

- doctor
- nurse

**Place of work:**

- Hospital
- Non-hospital health establishment

**Time working with patients with multimorbidity (2 or more chronic diseases):**

- less than or equal to 1 year
- greater than 1 year and less than or equal to 5 years
- greater than 5 years and less than or equal to 10 years
- greater than 10 years

**How many patients with multimorbidity do you think you see in a typical work week?**

**Portuguese version of MMD-HP**

**Moral Distress Measurement – ​​Health Professionals**

Moral distress occurs when professionals cannot perform what they believe to be ethically appropriate actions due to constraints or barriers. This survey presents situations that occur in clinical practice. If you have ever experienced these situations, they may or may not have been morally difficult. Please indicate how often you have experienced the situation described in each item and quantify how much moral distress it caused you. If you have never experienced a given situation, select "0" (never) for the frequency. Even if you have not experienced the situation, please indicate how much moral distress it would have caused you if it had occurred in your practice.

**Note that you will respond to each item, marking your response in both dimensions: Frequency and Level of Distress.**

|  | **Frequency** | | | | | **Level of Suffering** | | | | |
| --- | --- | --- | --- | --- | --- | --- | --- | --- | --- | --- |
|  | Never Very  often | | | | | None Very  intense | | | | |
|  | 0 | 1 | 2 | 3 | 4 | 0 | 1 | 2 | 3 | 4 |
| 1.Witnessing healthcare professionals giving “false hope” to a patient or family. |  |  |  |  |  |  |  |  |  |  |
| 2.Continuing aggressive treatment at the family's insistence, even if it is not believed to be in the patient's best interest. |  |  |  |  |  |  |  |  |  |  |
| 3.Feeling pressured to request or comply with a request for tests and treatments that you consider unnecessary or inappropriate. |  |  |  |  |  |  |  |  |  |  |
| 4.Being unable to provide the best possible care  due to pressure from management or insurers to reduce costs. |  |  |  |  |  |  |  |  |  |  |
| 5. Continuing to provide aggressive treatment to a person who will most likely die regardless of this treatment, when no one makes the decision to stop it. |  |  |  |  |  |  |  |  |  |  |
| 6. Being pressured not to act when I see that a doctor, nurse, or other staff member has made a medical error and  does not report it. |  |  |  |  |  |  |  |  |  |  |
| 7.Being asked to care for patients when I don't feel qualified to do so. |  |  |  |  |  |  |  |  |  |  |
| 8.Participating in care that causes unnecessary suffering or does not adequately relieve pain or other symptoms. |  |  |  |  |  |  |  |  |  |  |
| 9.Observe patient care being affected due to lack of continuity. |  |  |  |  |  |  |  |  |  |  |

| 10.Comply with a doctor or family member's request not to discuss the prognosis with the patient/family. |  |  |  |  |  |  |  |  |  |  |
| --- | --- | --- | --- | --- | --- | --- | --- | --- | --- | --- |
| 11.Witnessing a violation of a standard of professional practice or ethical principles and not feeling sufficiently supported to report it. |  |  |  |  |  |  |  |  |  |  |
| 12.Participating in care that I do not agree with, but having to do so for fear of litigation. |  |  |  |  |  |  |  |  |  |  |
| 13.I will be required to work with other members of the healthcare teamwho are not as competent as patient care requires. |  |  |  |  |  |  |  |  |  |  |
| 14.Witnessing poor quality patient care due to poor team communication. |  |  |  |  |  |  |  |  |  |  |
| 15. Feeling pressured to ignore situations in which patients have not been given sufficient information to ensure informed consent. |  |  |  |  |  |  |  |  |  |  |
| 16.Being asked to care for more patients than I can safely care for. |  |  |  |  |  |  |  |  |  |  |
| 17.Experience compromised patient care due to lack of resources/equipment/bed capacity. |  |  |  |  |  |  |  |  |  |  |
| 18.Experiencing a lack of action or administrative support for a problem that compromises patient care. |  |  |  |  |  |  |  |  |  |  |
| 19.Feeling that patient care is compromised by excessive bureaucracy. |  |  |  |  |  |  |  |  |  |  |
| 20.Fear reprisals if you speak frankly. |  |  |  |  |  |  |  |  |  |  |
| 21.Feeling insecure/intimidated/bullied by my own colleagues. |  |  |  |  |  |  |  |  |  |  |
| 22. Having to work with abusive patients/family members who compromise the quality of care. |  |  |  |  |  |  |  |  |  |  |
| 23.Feeling obliged to overvalue tasks and measures of productivity or quality to the detriment of patient care. |  |  |  |  |  |  |  |  |  |  |
| 24.Having to care for patients who have ambiguous or inconsistent treatment plans or who do not have defined goals of care. |  |  |  |  |  |  |  |  |  |  |
| 25.Working within power hierarchies within my team, unit, or institution that compromise patient care. |  |  |  |  |  |  |  |  |  |  |
| 26.Being part of a team that conveys inconsistent messages to the patient/family. |  |  |  |  |  |  |  |  |  |  |
| 27.Working with team members who do not treat vulnerable or stigmatized patients with dignity and respect. |  |  |  |  |  |  |  |  |  |  |
| If there are other situations in which you have felt moral distress, please write them down and rate them here: |  |  |  |  |  |  |  |  |  |  |
|  |  |  |  |  |  |  |  |  |  |  |
|  |  |  |  |  |  |  |  |  |  |  |

**Have you ever left or considered leaving your position due to moral distress?**

- No, I never did nor considered doing so.
- Yes, I considered doing it, but I didn't.
- Yes, I have.

**Are you considering leaving your position due to moral distress?**

- Yes
- No

**Below are five statements with which you may agree or disagree. Using the 1-7 scale below, indicate your degree of agreement with each item by placing the appropriate number on the line preceding each item. Be honest in your responses. Here's the 7-point scale:**

| 1 – totally disagree |  | | | | | | |
| --- | --- | --- | --- | --- | --- | --- | --- |
| 2 – in disagreement |  |  |  |  |  |  |  |
| 3 – more or less in disagreement |  |  |  |  |  |  |  |
| 4 – neither in agreement nor in disagreement |  |  |  |  |  |  |  |
| 5 – more or less agree |  |  |  |  |  |  |  |
| 6 – agreed |  |  |  |  |  |  |  |
| 7 – I totally agree |  |  |  |  |  |  |  |
| 1. In many ways, my life comes close to my ideals. | 1 | 2 | 3 | 4 | 5 | 6 | 7 |
| 2. My living conditions are excellent. | 1 | 2 | 3 | 4 | 5 | 6 | 7 |
| 3. I am satisfied with my life. | 1 | 2 | 3 | 4 | 5 | 6 | 7 |
| 4. So far, I have managed to achieve what is important in life. | 1 | 2 | 3 | 4 | 5 | 6 | 7 |
| 5. If I could live my life over again, I wouldn't change anything. | 1 | 2 | 3 | 4 | 5 | 6 | 7 |

**Thank you for participating.**
