# Supplementary material for: Caring for patients with multimorbidity: moral distress and life satisfaction among doctors and nurses in Portugal
Source: PeerJ. 2026 Apr 29;14:e21230. doi: 10.7717/peerj.21230 (PMC13135333; doi:10.7717/peerj.21230)
Supplement: Supplemental Information 5 [file peerj-14-21230-s005.pdf]

```
CODEBOOK Sex [n] AgeYears [s] Age_cat [n] maritalstatus [n] professionalrole [n]
workplace [n] numberyearsworkingpatientsmultimorbidity [n]
numberpatientsmultimorbidityseenperweek [s] Multimorbidityclinicalworkload [n]
Haveyoueverleftorconsideredleaving [n] Areyouconsideringleavingyourpositionnow [n] MD
[s] SWLS [s]
/VARINFO LABEL VALUELABELS
/OPTIONS VARORDER=VARLIST SORT=ASCENDING MAXCATS=200
/STATISTICS NONE.
```

Codebook

Notes

Contents		
Output Created		19-SEP-2025 01:24:47
Comments		
Input	Data	C:\Users\user\Desktop\Submissoes\Sofrimento_moral\Artigo\FINAL\English_Base_dados_SPSS.sav
	Active Dataset	DataSet1
	Filter	<none>
	Weight	<none>
	Split File	<none>
	N of Rows in Working Data File	340
Syntax		CODEBOOK Sex [n] AgeYears [s] Age_cat [n] maritalstatus [n] professionalrole [n] workplace [n] numberyearsworkingpatientsmultimorbidity [n] numberpatientsmultimorbidityseenperweek [s] Multimorbidityclinicalworkload [n] Haveyoueverleftorconsideredleaving [n] Areyouconsideringleavingyourpositionnow [n] MD [s] SWLS [s] /VARINFO LABEL VALUELABELS /OPTIONS VARORDER=VARLIST SORT=ASCENDING MAXCATS=200 /STATISTICS NONE.
Resources	Processor Time	0 00:00:00,02
	Elapsed Time	0 00:00:00,00

```
[DataSet1] C:-
\Users\user\Desktop\Submissoes\Sofrimento_moral\Artigo\FINAL\English_Base_dados_SPSS.sav
```

Sex

		Value
Standard Attributes	Label	<none>
Valid Values	1	male
	2	female

AgeYears

		Value
Standard Attributes	Label	<none>

Age_cat

		Value
Standard Attributes	Label	<none>
Valid Values	1	Under 35
	2	35 to 50
	3	Over 50

maritalstatus

		Value
Standard Attributes	Label	<none>
Valid Values	1	Married/or in a stable relationship
	2	Single/Separated/Divorced/Widowed

professionalrole

		Value
Standard Attributes	Label	<none>
Valid Values	1	doctor
	2	nurse

workplace

		Value
Standard Attributes	Label	<none>
Valid Values	1	Hospital
	2	Non-hospital healthcare facility

numberyearsworkingpatientsmultimorbidity

		Value
Standard Attributes	Label	<none>
Valid Values	1	10 or less
	2	More than 10

numberpatientsmultimorbidityseenperweek

		Value
Standard Attributes	Label	<none>

Multimorbidityclinicalworkload

		Value
Standard Attributes	Label	<none>
Valid Values	0	High
	1	Low

Haveyoueverleftorconsideredleaving

		Value
Standard Attributes	Label	Have you ever left or considered leaving a clinical position due to moral distress?
Valid Values	1	No, I have never considered leaving or left a position
	2	Yes, I considered leaving but did not leave
	3	Yes, I left a position

Areyouconsideringleavingyourpositionnow

		Value
Standard Attributes	Label	Are you considering leaving your position now due to moral distress?
Valid Values	1	No
	2	Yes

MD

		Value
Standard Attributes	Label	MD (MMD-HP)

SWLS

		Value
Standard Attributes	Label	<none>
